# Cold plasma as a dual strategy for insect control and seed quality enhancement in chickpea, lentil, and cereal by-products

**DOI:** 10.1038/s41598-026-41194-y

**Published:** 2026-04-20

**Authors:** Mahdiyeh Martami, Mohamad Sajad Nikouei Fard, Maryam Abouheydari, Amir Hossein Abbasi, Mohammad Hossein Mohajer, Najmeh Ebrahimi, Aidin Hamidi, Hamid Reza Ghomi

**Affiliations:** 1 Laser and Plasma Research Institute, Shahid Beheshti University, Tehran, 1983969411 Iran; 2 Department of Animal, Marine and Aquatic Biology and Biotechnology, Faculty of Life Sciences and Biotechnology, Shahid Beheshti University, Tehran, Iran; 3 Entomology Research Department, Iranian Research Institute of Plant Protection, Agricultural Research, Education and Extension Organization (AREEO), Tehran, Iran; 4 Seed and Plant Certification and Registration Institute (SPCRI), Agriculture Research, Education and Extension Organization (AREEO), Karaj, Iran

**Keywords:** Non-thermal plasma, Pest management, Agriculture, *Callosobruchus maculatus*, *Tribolium castaneum*, Surface modification, Plant sciences, Zoology

## Abstract

Cold plasma is a sustainable, chemical-free technology for pest control and seed enhancement. This study evaluated the biological effects of dielectric barrier discharge (DBD) plasma on two stored-product pests and legume seeds. Various life stages were exposed to targeted plasma treatments: for Callosobruchus maculatus, adults and eggs were treated at 20–24 kV for 1 to 9 min, while for *Tribolium castaneum*, larvae, pupae and adults were exposed to 13–23 kV for 5–120 s. For *T. castaneum*, 100% mortality was achieved: larvae at 19 kV for 30 s, adults at 23 kV for 30 s, and pupae at 23 kV for 90 s. Mortality in other samples increased only slightly (2–5%) after 24 h, showing the effect is primarily immediate. For *C. maculatus*, immediate mortality exceeded 40% in all treatments and reached 100% at 24 kV for ≥ 5 min, with a > 10% increase after 24 h. Adult emergence was suppressed entirely at 24 kV for 9 min. Beyond insecticidal effects, plasma improved seed performance. In chickpea (Saeed and Mansur varieties), germination rate increased by over 20%, with seedling vigor and root/shoot growth also growing more than 20%. Lentils showed a 3% germination increase and 11–12% improvements in root and shoot length. Seed biomass increased, with fresh/dry weight gains of 12%/7% in Saeed, 25%/19% in Mansur, and ~ 6% in lentils. Water uptake rose dramatically by 75% in Saeed, 85% in Mansur, and 70% in lentils. FTIR spectra confirmed higher surface polarity (stronger O-H/N-H bands), and SEM images revealed seed coat etching and micro-cracks, explaining the improved wettability. These results demonstrate the dual functionality of cold plasma as a sustainable postharvest technology, effectively controlling pests and enhancing seed quality.

## Introduction

Global food security is continually threatened by significant losses caused by insect pests that infest stored grains and food products. Species such as flour beetles (*Tribolium spp*.) are among the most destructive, leading to qualitative and quantitative deterioration of cereals and processed products during storage^1^. In particular, the genus Tribolium, including the red flour beetle( *Tribolium castaneum*- *Coleoptera: Tenebrionidae*,* Herbst*,* 1797* ) and the confused flour beetle(*Tribolium confusum -Coleoptera: Tenebrionidae*,* Jacquelin du Val*,* 1868* )is a major cosmopolitan pest responsible for considerable economic losses^2^. Both larvae and adults are generalist feeders capable of attacking almost any dried plant or animal material, with cereal and cereal-based products being especially vulnerable. Infestation often results in product contamination with larval skins and pupae, and secretions of benzoquinones from the insects’ abdominal glands can produce a persistent, unpleasant odor^3^. Similarly, bruchid beetles (*Callosobruchus spp*.) are primary pests of stored legumes such as chickpeas. Infestation by *Callosobruchus maculatus (Coleoptera: Chrysomelidae: Bruchinae -Fabricius*,* 1775)* frequently begins in the field before harvest. Each emerging adult consumes approximately 25% of the seed from which it appears, significantly reducing seed quality and often preventing germination. Heavy infestations can also raise the temperature of stored products, promoting mold growth and further degrading product quality^4^. These pests are particularly problematic in tropical and subtropical regions, where favorable environmental conditions, inadequate storage methods, and proximity of agricultural and storage facilities contribute to increased infestation risks^5^.

Conventional pest management has primarily relied on chemical insecticides, including contact and fumigants such as methyl bromide and phosphine, applied during crop growth and storage^6^. However, the extensive use of these chemicals has raised serious concerns, including environmental contamination^7^, non-target toxicity^8^, risks to human health^9^, and the development of insecticide resistance in pest populations^10^. These drawbacks underscore the urgent need for novel, sustainable, and eco-friendly alternatives for insect pest control in stored food products.

In this context, researchers have explored a variety of alternative methods, including biological control (e.g., predators, parasitoids, and entomopathogenic organisms)^11,12^, botanical pesticides^13,14^, Varietal Resistance^15,16^, integrated pest management strategies^17^ and physical methods such as heating^18,19^, and modified atmospheres^20,21^. Cold plasma technology has recently emerged as a promising physical control method for storing insect pests. Cold plasma generates a range of reactive species, including UV photons, free radicals, ions, and electrons at ambient temperature, with minimal thermal impact on treated products^22^. Numerous studies have demonstrated the efficacy of cold plasma in decontaminating grains^23^, promoting seed germination^24^, and eliminating insect pests in stored foods^25,26^. Cold plasma employs multiple mechanisms, including oxidative stress, etching effects, and electromagnetic interactions to induce mortality and sublethal effects in insect life stages^27^. The reactive oxygen and nitrogen species generated can cause oxidative damage to proteins, lipids, and DNA, ultimately leading to cellular disruption and insect death^28^. The charged particles and UV radiation may also contribute to the multi-trophic mode of action.

This study evaluates the insecticidal potential of cold plasma treatment against two major stored-product pests—red flour beetle *(T. castaneum)* and pulse beetle *(C. maculatus)* —using chickpeas and rolled oats as representative substrates. The influence of plasma exposure parameters- including treatment duration, applied voltage, and post-treatment preservation time was assessed for their effects on mortality across developmental stages (eggs, larvae, pupae, and adults). In addition, seed quality was evaluated through germination tests measuring germination percentage, germination speed, shoot and root length, and wet and dry biomass, which were conducted on all samples. In addition to these tests, contact angles, SEM, and ATR-FTIR analysis were conducted on samples treated at the optimal voltage and time (to control *Callosobruchus maculatus)*, to assess surface degradation and hydrophilicity. ATR-FTIR analysis was also performed on flour, bran, and rolled oats to evaluate plasma-induced surface modifications.

Overall, this study aims to establish cold plasma as a sustainable, scalable alternative to conventional insecticide-based strategies, ensuring long-term food security by mitigating the challenges posed by insect infestations during storage without adversely affecting the environment or human health.

## Materials and methods

### Plasma

**Fig. 1:**
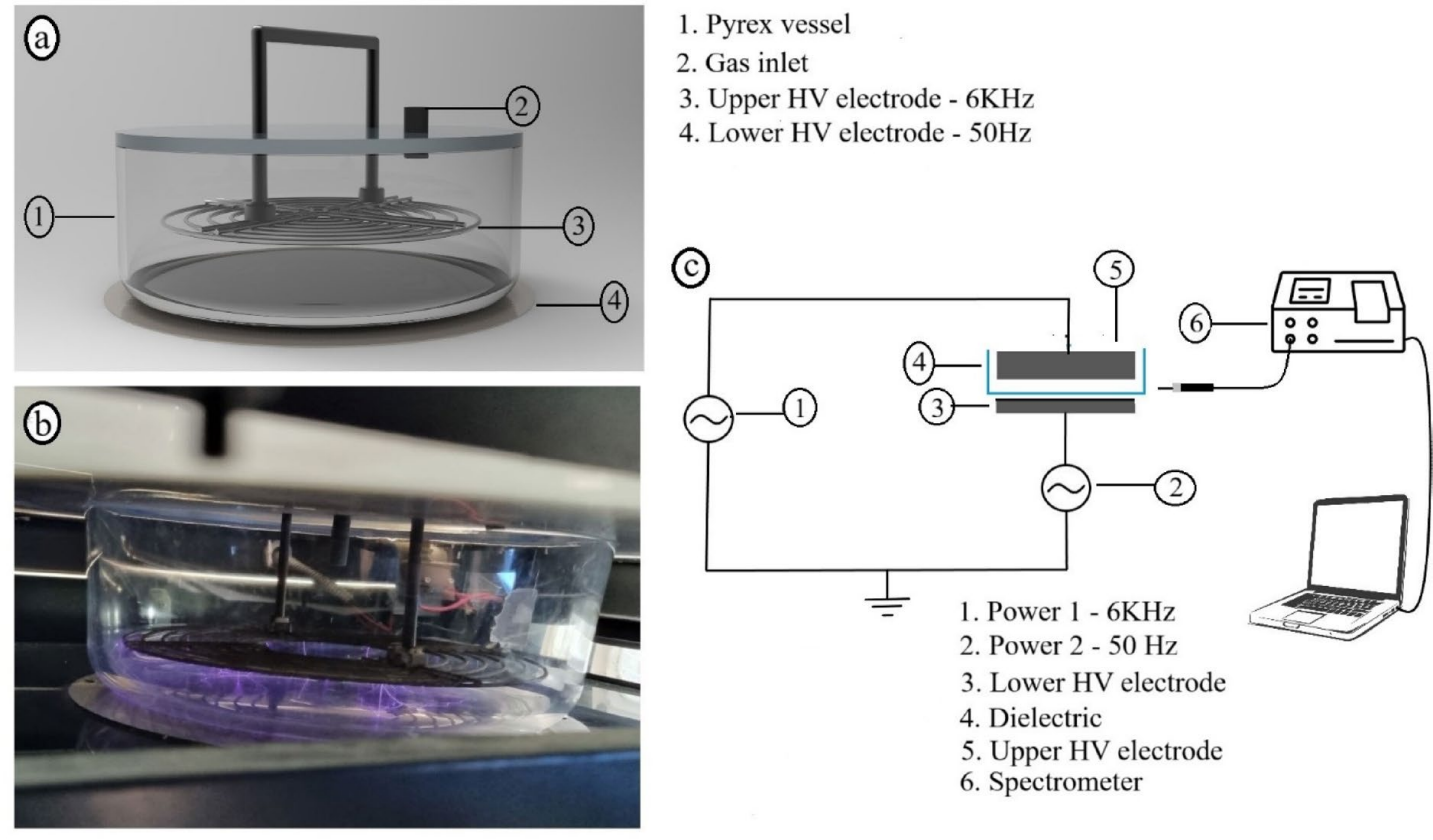
Schematic and photographic overview of the low-pressure plasma reactor setup. (**a**) A computer-aided design (CAD) model of the plasma chamber created using SolidWorks. (**b**) Photograph of the actual plasma chamber in operation, showing the generated plasma. (**c**) A schematic diagram of the complete experimental setup, detailing the overall system configuration.

Cold plasma was generated using a dielectric barrier discharge (DBD) system (Enhancedtech-151, Kavosh Yaran Fan Pooya Co., Iran). A detailed schematic, including a circuit diagram and a photograph of the experimental setup, is presented in Fig. 1.

The system consisted of two asymmetric stainless-steel electrodes operating with different electrical parameters. The upper electrode was powered by a 6 kHz pulsed direct current source with an adjustable output voltage of 10 to 24 kV, while the lower electrode operated at 50 Hz with a voltage range of 0 to 24 kV. A cylindrical Pyrex vessel (diameter: 22 cm; height: 10 cm; wall thickness: 4 mm) served as a dielectric barrier between the electrodes, with a 1.5 cm spacing.

#### *C.maculatus*

##### Insect rearing conditions and sample preparation

Colonies of C. maculatus were maintained in 1200 mL plastic containers (10 × 7 × 18 cm) fitted with a 12 × 7 cm ventilation opening covered with fine-mesh silk cloth to ensure adequate ventilation while preventing insect escape. The insects were reared on a mixed substrate of chickpeas and mung beans. Initial colony establishment began with 20 insects obtained from infested market products, allowing uninterrupted reproduction for 6 months to develop robust populations.

All chickpeas used in the study were previously sterilized by freezing at -18 °C for 48 h to eliminate any pre-existing infestation. These sterilized chickpeas were introduced into established colonies specifically to serve as an egg-laying substrate. After 24 h of exposure to the breeding colony, the egg-laden chickpeas were transferred to new containers for further development.

For adult insect assays, emerging adults were collected and precisely aged, and 3-day-old adults were selected for all experiments. For egg assays, chickpeas with 24-hour-old eggs were used directly. This systematic approach of transferring chickpeas to new containers after each egg-laying cycle ensured the production of age-synchronized insects for standardized testing. Each experimental replicate consistently used 10 adult insects or 10 eggs, maintaining uniformity across all treatments.

##### Exposure of tested insects to cold plasma

For plasma treatment, a standardized mass of 135 g of chickpeas was first placed inside the dielectric barrier discharge (DBD) chamber. Subsequently, 10 three-day-old adult insects were introduced as test samples. Chickpeas bearing exactly 10 eggs (24-hours-old) distributed across five chickpeas. All treatments were conducted at atmospheric pressure. The tested voltages were 20, 22, and 24 kV, each applied for 1, 3, 5, 7, and 9 min, respectively. Each unique combination of voltage and exposure time was replicated five times. For each replication, corresponding control samples (10 insects or 10 eggs) were randomly selected and processed identically, except without plasma exposure.

Following treatment, all samples were transferred to 240 mL containers (10 × 3 × 8 cm) equipped with a 3 × 3 cm mesh-covered ventilation opening and incubated in a dark environment at 27 °C and 60% relative humidity^1,29^.

#### *T. castaneum*

##### Insect rearing conditions and sample preparation

A mixture of flour, rolled oats, and bran was used for rearing *T. castaneum*. Fifty initial insects were obtained from the Iranian Research Institute of Plant Protection and allowed to reproduce for six months to establish a stable colony. The colonies were maintained in 1200 mL plastic containers (10 × 7 × 18 cm) with a mesh-covered vent, identical to those used for C. maculatus.

For colony maintenance, approximately 400–500 unsorted adult insects were placed in flour, which was then stored at − 18 °C for 48 h to promote assimilation. After this period, the flour containing the adults was sieved through a 1 mm sieve to remove the adult insects. The remaining flour, which contained insect eggs, was mixed with equal portions of bran and rolled oats and returned to the incubator. To ensure age-synchronized test subjects for experiments, this rearing and separation process was repeated for each specific life stage.

For plasma treatment, samples were prepared by placing 10 individuals of specific life stages —20-day-old larvae, 25–27-day-old pupae, or 3-day-old adults — into 100 g of rolled oats, which served as the substrate for the plasma exposure experiments.

##### Exposure of tested insects to cold plasma

Plasma treatment was performed at atmospheric pressure using a comprehensive experimental matrix. Insects at each life stage (larvae, pupae, and adults) were exposed to six voltage levels (13, 15, 17, 19, 21, and 23 kV) across five exposure durations (5, 30, 60, 90, and 120 s). Each unique combination of life stage, voltage, and exposure time was replicated five times.

For each replication, control samples consisting of 10 insects at the corresponding life stage were randomly selected and handled identically, except without plasma exposure. Following treatment, all samples (both treated and control) were preserved in 240 mL containers (10 × 3 × 8 cm). These containers were fitted with a 3 × 3 cm ventilation opening covered with fine mesh silk cloth to ensure adequate aeration. Following the identification of optimal lethal parameters from these tests, the efficacy of plasma treatment was further verified using flour and bran as alternative substrates at the determined optimum voltage and exposure time.

All colonies and test samples, both during and after treatment, were maintained under controlled incubation conditions at 27 °C and 60% relative humidity in complete darkness^1,29^.

### Plasma seed treatment and germination assay

#### Plant materials and plasma treatment

Seeds of two chickpea cultivars (Cicer arietinum L. cvs. Saeed and Mansur) and one lentil cultivar (Lens culinaris Medik. cv. Bileh Sawar) were obtained from the Iranian Seed and Plant Certification and Registration Institute. Plasma treatment was conducted using the same dielectric barrier discharge (DBD) system described in Sect.  2.1. Seeds were exposed to cold plasma under the same experimental conditions as for C. *maculatus* treatments, with voltages of 20, 22, and 24 kV and exposure durations of 1, 3, 5, 7, and 9 min. Each treatment combination was replicated three times, with each replicate consisting of 20 seeds. Control groups for each cultivar underwent identical handling procedures without plasma exposure.

#### Germination test and seedling evaluation

Germination tests for chickpea and lentil seeds were conducted following the standard procedures outlined by the International Seed Testing Association (ISTA, 2023) with slight modifications for each species to accommodate their specific physiological requirements^30^.

For Chickpea Seeds (Saeed and Mansur varieties), 20 seeds from each replicate were placed between two layers of moist filter paper (Whatman No. 1) in sterilized 9 cm cantainers. The containers were incubated in a growth chamber at a constant temperature of 20 ± 1 °C under a 12-hour light/12-hour dark photoperiod. Germinated seeds (with a radicle protrusion of ≥ 2 mm) were counted daily for a period of 7 days. The final germination count and the daily counts for germination rate calculation were recorded on the 7th day.

For Lentil Seeds (Bileh Sawar variety), the same setup was used as for chickpeas (20 seeds per replicate on moist filter paper in Petri dishes). However, to account for the potentially different germination kinetics of lentils, the incubation period was extended. Germinated seeds were counted daily for 10 days, with the final evaluation performed on the 10th day.

After the final germination count (day 7 for chickpea and day 10 for lentil), five vigorous and normal seedlings from each replicate were randomly selected for biometric measurements. Root and shoot lengths were measured to the nearest centimeter. For biomass determination, the fresh weight of these seedlings was recorded immediately. Subsequently, the seedlings were oven-dried at 80 °C for 24 h to obtain their constant dry weight, measured using a digital analytical balance.

**Table 1:** Summary of experimental design parameters for cold plasma treatments.

Sample type/species	Variety/stage	Substrate	Voltage (kV)	Exposure time	Replicates	Sample size per replicate
*C. maculatus*	Adult (3-day-old)	135 g chickpeas	20, 22, 24	1, 3, 5, 7, 9 min	5	10 insects
	Egg (24-hour-old)	5 chickpeas	20, 22, 24	1, 3, 5, 7, 9 min	5	10 eggs
*T. castaneum*	Larva (20-day-old)	100 g rolled oats	13, 15, 17, 19, 21, 23	5, 30, 60, 90, 120 s	5	10 larvae
	Pupa (25-27-day-old)	100 g rolled oats	13, 15, 17, 19, 21, 23	5, 30, 60, 90, 120 s	5	10 pupae
	Adult (3-day-old)	100 g rolled oats	13, 15, 17, 19, 21, 23	5, 30, 60, 90, 120 s	5	10 adults
Chickpea	Saeed	–	20, 22, 24	1, 3, 5, 7, 9 min	3	20 seeds
	Mansur	–	20, 22, 24	1, 3, 5, 7, 9 min	3	20 seeds
Lentil	Bileh Sawar	–	20, 22, 24	1, 3, 5, 7, 9 min	3	20 seeds

### Insect analysis

#### Measuring insect mortality

##### *C.maculatus*

For adult insect mortality, individuals were counted immediately after treatment and again after 24 h. The percentage of dead insects was calculated using the following formula:

1 $$\text{Insect's mortality percentage} = \:\frac{\left(\:\mathrm{n}\mathrm{u}\mathrm{m}\mathrm{b}\mathrm{e}\mathrm{r}\:\mathrm{o}\mathrm{f}\:\mathrm{d}\mathrm{e}\mathrm{a}\mathrm{d}\:\mathrm{i}\mathrm{n}\mathrm{s}\mathrm{e}\mathrm{c}\mathrm{t}\mathrm{s}\:\times\:100\right)}{\left(\mathrm{t}\mathrm{o}\mathrm{t}\mathrm{a}\mathrm{l}\:\mathrm{n}\mathrm{u}\mathrm{m}\mathrm{b}\mathrm{e}\mathrm{r}\:\mathrm{o}\mathrm{f}\:\mathrm{i}\mathrm{n}\mathrm{s}\mathrm{e}\mathrm{c}\mathrm{t}\mathrm{s}\right)}$$

The percentage of adult emergence was calculated using “Eq. (2) “. The number of insects emerging from treated eggs was monitored daily for fourteen days after the first appearance.

2 $$\text{Insect's emergence percentage} =\:\frac{\left(\mathrm{t}\mathrm{h}\mathrm{e}\:\mathrm{n}\mathrm{u}\mathrm{m}\mathrm{b}\mathrm{e}\mathrm{r}\:\mathrm{o}\mathrm{f}\:\mathrm{i}\mathrm{n}\mathrm{s}\mathrm{e}\mathrm{c}\mathrm{t}\mathrm{s}\:\times\:100\right)}{\left(\mathrm{t}\mathrm{h}\mathrm{e}\:\mathrm{t}\mathrm{o}\mathrm{t}\mathrm{a}\mathrm{l}\:\mathrm{n}\mathrm{u}\mathrm{m}\mathrm{b}\mathrm{e}\mathrm{r}\:\mathrm{o}\mathrm{f}\:\mathrm{e}\mathrm{e}\mathrm{g}\mathrm{s}\right)}$$

##### *T. castaneum*

We evaluated *T. castaneum* mortality across different life stages. We assessed pupal mortality 48 h after treatment using “Eq. (3)”.

3 $$\text{Pupae ' s mortality percentage} =\:\frac{\left(\mathrm{n}\mathrm{u}\mathrm{m}\mathrm{b}\mathrm{e}\mathrm{r}\:\mathrm{o}\mathrm{f}\:\mathrm{d}\mathrm{e}\mathrm{a}\mathrm{d}\:\mathrm{p}\mathrm{u}\mathrm{p}\mathrm{a}\mathrm{e}\:\times\:100\right)}{\left(\mathrm{t}\mathrm{o}\mathrm{t}\mathrm{a}\mathrm{l}\:\mathrm{n}\mathrm{u}\mathrm{m}\mathrm{b}\mathrm{e}\mathrm{r}\:\mathrm{o}\mathrm{f}\:\mathrm{p}\mathrm{u}\mathrm{p}\mathrm{a}\mathrm{e}\right)}$$

To count adults and larvae, we spread treated oats or bran in a container for manual inspection, while we sieved flour samples (500 μm). We counted dead individuals immediately and 24 h post-treatment, calculating the mortality percentages for adults and larvae with “Eqs. (1) and (4) “, respectively.

4 $$\text{Larvae' s mortality percentage} =\:\frac{\left(\mathrm{n}\mathrm{u}\mathrm{m}\mathrm{b}\mathrm{e}\mathrm{r}\:\mathrm{o}\mathrm{f}\:\mathrm{d}\mathrm{e}\mathrm{a}\mathrm{d}\:\mathrm{l}\mathrm{a}\mathrm{r}\mathrm{v}\mathrm{a}\mathrm{e}\:\:\times\:100\right)}{\left(\mathrm{t}\mathrm{o}\mathrm{t}\mathrm{a}\mathrm{l}\:\mathrm{n}\mathrm{u}\mathrm{m}\mathrm{b}\mathrm{e}\mathrm{r}\:\mathrm{o}\mathrm{f}\:\mathrm{l}\mathrm{a}\mathrm{r}\mathrm{v}\mathrm{a}\mathrm{e}\right)}\:$$

#### Examination of plasma’s morphological effects on the insect Body

To assess the impact of plasma on insect morphology, 20 treated adults (exposed at optimal conditions) and 20 untreated controls were analyzed. Specimens were anesthetized using chloroform and fixed on a paraffin bed. Heads were dissected using a No. 11 scalpel under a stereomicroscope, and brain tissues were examined for visible damage.

### Seed germination analysis

Germination parameters were quantified to evaluate the effects of plasma treatment on seed performance. Germination percentage was calculated using “Eq. (5)”, where N_i_ is the number of germinated seeds and N is the total number of seeds.

5 $${\text{Germination Percentage }} = {\text{ }}({\mathrm{N}}_{{\mathrm{i}}} \times {\text{ 1}}00)/\left( {\mathrm{N}} \right)$$

Germination rate was determined using “Eq. (6) “:

6 $${\text{Germination Rate }} = {\text{ }}\sum {\text{ n}}_{{\mathrm{i}}} /{\mathrm{d}}_{{\mathrm{i}}}$$

In this case, n_i_ is the number of germinated seeds, and d_i_ is the number of days^31^.

Following the 10-day incubation period, seedling growth parameters were assessed. Five vigorous seedlings from each replicate were randomly selected for morphological and biomass measurements. Root and shoot lengths were measured to the nearest centimeter. For biomass determination, fresh weight was measured immediately, while dry weight was obtained after oven-drying at 80 °C for 24 h, using a digital laboratory scale.

### The effect of plasma on the substance’s surface

Fourier-transform infrared (FTIR) spectroscopy, water droplet contact angle measurements, and Scanning Electron Microscopy (SEM) were used to investigate physicochemical modifications in seeds and cereal by-products surfaces following cold plasma treatment at optimum voltage and time. The samples analyzed included two chickpea varieties (Saeed and Mansur) and lentil (Bileh Sawar). They were collected for both control and plasma-treated samples. Only the FTIR test was conducted for bran, rolled oats, and flour.

### Statistical analysis

All data were subjected to one-way analysis of variance (ANOVA). Means were separated using Duncan Honestly Significant Difference (HSD) post-hoc test at a significance level of *p* < 0.05. All statistical analyses were performed using IBM SPSS Statistics software (Version 27.0, IBM Corp., Armonk, NY, USA). Data in the text and tables are presented as mean ± standard error (SE).

## Results

### Insect mortality

#### *C.maculatus*

**Fig. 2:**
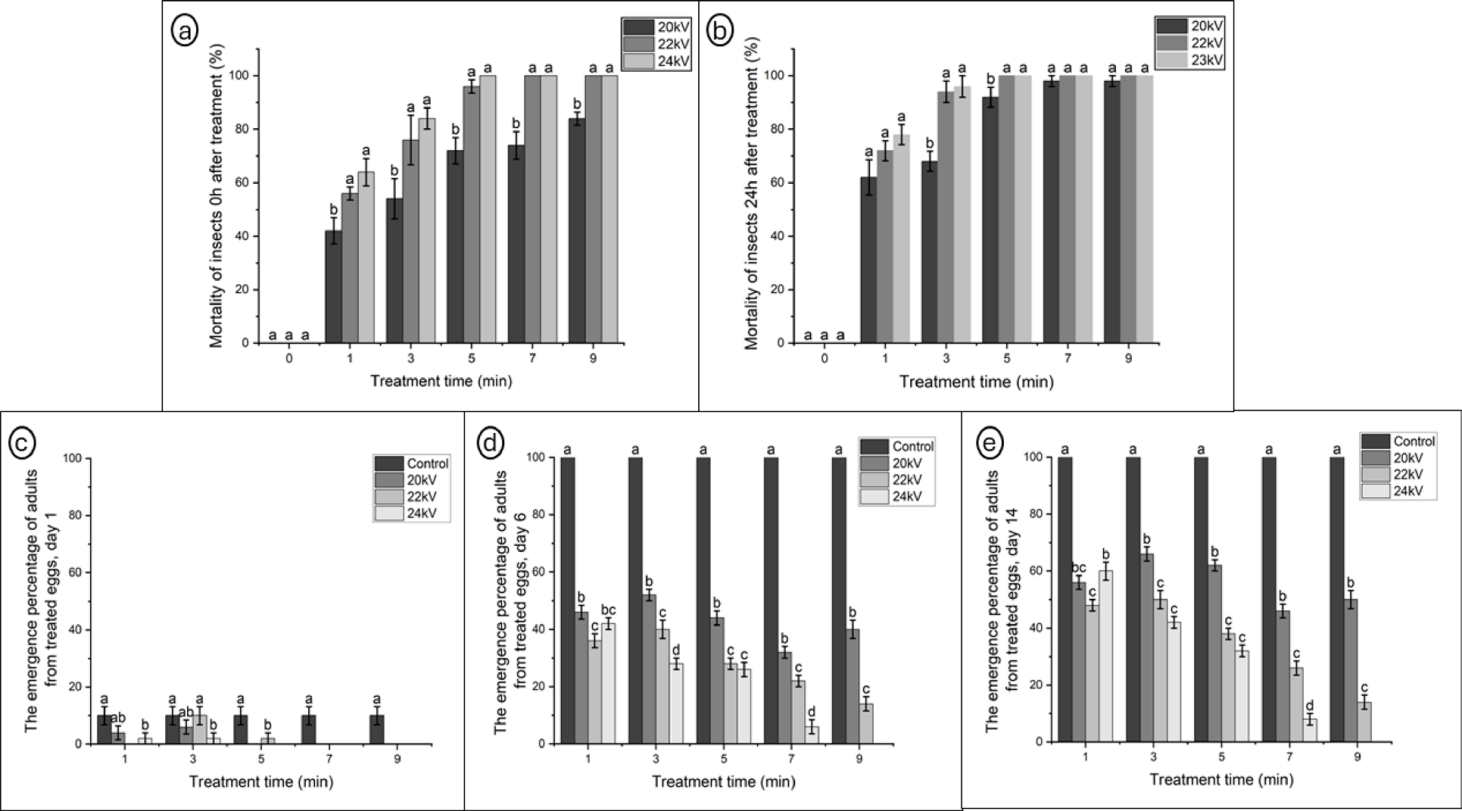
Effect of cold plasma treatment on adult mortality and adult emergence of *Callosobruchus maculatus*. (**a**) Mortality rate of adults immediately after treatment; (**b**) Mortality rate of adults 24 h after treatment; (**c**) Percentage of adult emergence from treated eggs on day 1; (**d**) Percentage of adult emergence from treated eggs on day 6; (**e**) Percentage of adult emergence from treated eggs on day 14. Data are presented as a mean value ± standard error. Different letters above bars indicate statistically significant differences among treatments according to one-way ANOVA (*p* < 0.001).

The effects of cold plasma exposure on adults’ mortality were evaluated immediately after treatment (Fig. 2a) and 24 h post-treatment (Fig. 2b). Immediately after treatment, mortality rate in all treated groups were significantly higher than in the control, exceeding 40% in all cases (*p* < 0.001). Complete adult mortality (100%) was achieved at 24 kV for exposure times of 5, 7, and 9 min, as well as at 22 kV for 7 and 9 min. These results indicate that exposure duration had a more pronounced effect on adult mortality than applied voltage. After 24 h, mortality increased significantly across all treatments, with rates exceeding 90% from 5 min onward at all voltage levels (*p* < 0.001), confirming the irreversible lethal effects of plasma exposure.

Adult emergence from plasma-treated eggs was first observed 28 days after treatment. On the first observation day, the control group and the 22 kV–3 min treatment showed the highest emergence rates (10 ± 3.16%), while emergence was limited or absent in the remaining treatments. By day 6, emergence in the control samples reached 100%, whereas the maximum emergence among treated samples was significantly lower, reaching only 46 ± 2.45% (*p* < 0.001). Notably, no insect emergence was observed at 24 kV for 9 min (Fig. 2d). After 14 days, plasma-treated samples exhibited a statistically significant reduction in insect emergence compared to the control (*p* < 0.001). At 20 kV, emergence remained relatively high (66 ± 2.44%), indicating the lowest treatment efficacy. In contrast, at 22 kV, emergence decreased progressively with increasing exposure time, from 50 ± 3.16% to 14 ± 2.45% after 9 min. A similar but more pronounced trend was observed at 24 kV, where emergence dropped below 10% after 7 min and was completely suppressed after 9 min. Overall, these results demonstrate that prolonged plasma exposure at higher voltages significantly inhibits egg hatchability, with the most effective condition being 24 kV for 9 min, under which *C. maculatus* emergence was entirely prevented.

#### *T. castaneum*

**Fig. 3:**
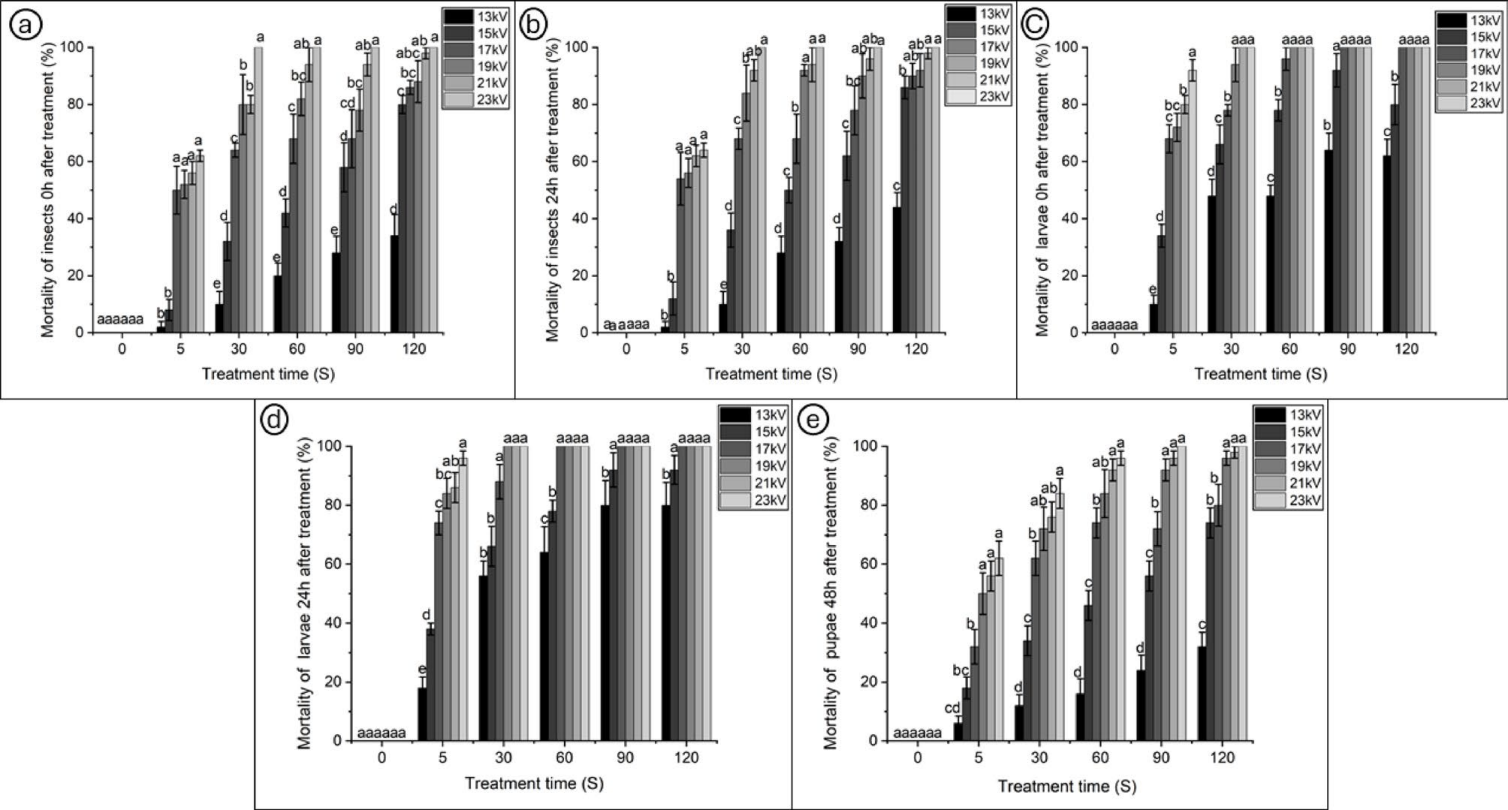
Effect of cold plasma treatment on adult and immature stages of *Tribolium castaneum*. (**a**) Mortality rate of adults immediately after treatment; (**b**) Mortality rate of adults 24 h after treatment; (**c**) Mortality rate of larvae immediately after treatment; (**d**) Mortality rate of larvae 24 h after treatment; (**e**) Mortality rate of pupae 48 h after treatment. Data are presented as a mean value ± standard error. Different letters above bars indicate statistically significant differences among treatments according to one-way ANOVA (*p* < 0.001).

The mortality response of *T. Castaneum* to cold plasma exposure was assessed across three developmental stages: larvae, pupae, and adults. Mortality was recorded immediately and 24–48 h post-treatment to capture acute and delayed effects (Fig. 3a–e). Plasma treatment resulted in statistically significant mortality in all life stages compared to the control (*p* < 0.001). In adult beetles (Fig. 3a, b), mortality increased significantly with increasing exposure time and voltage. Complete mortality (100%) was achieved at 23 kV after only 30 s of exposure. After 24 h, an additional 2–5% increase in mortality was observed in some treatments, a significantly lower delayed effect compared to that observed in *C. maculatus* adults (*p* < 0.001).

Larvae exhibited significantly higher sensitivity to plasma treatment than the other developmental stages. Even at the shortest exposure time (5 s), mortality exceeded 10% at all tested voltages, increasing significantly from 10 ± 3.16% at 13 kV to 92 ± 3.74% at 23 kV (*p* < 0.001), demonstrating a strong dose-dependent response.

Pupal mortality, evaluated 48 h post-treatment (Fig. 3e), showed a moderate but statistically significant increase compared to controls (*p* < 0.001), with mortality ranging from 60 ± 5.83% at 23 kV for 5 s to 100% at 23 kV for 120 s. This developmental stage exhibited the greatest resistance to plasma exposure than larvae and adults, with the first complete mortality recorded at 23 kV after 90 s, a longer exposure time than required for larvae or adults.

#### Morphological alterations in insect head structures following cold plasma exposure

**Fig. 4:**
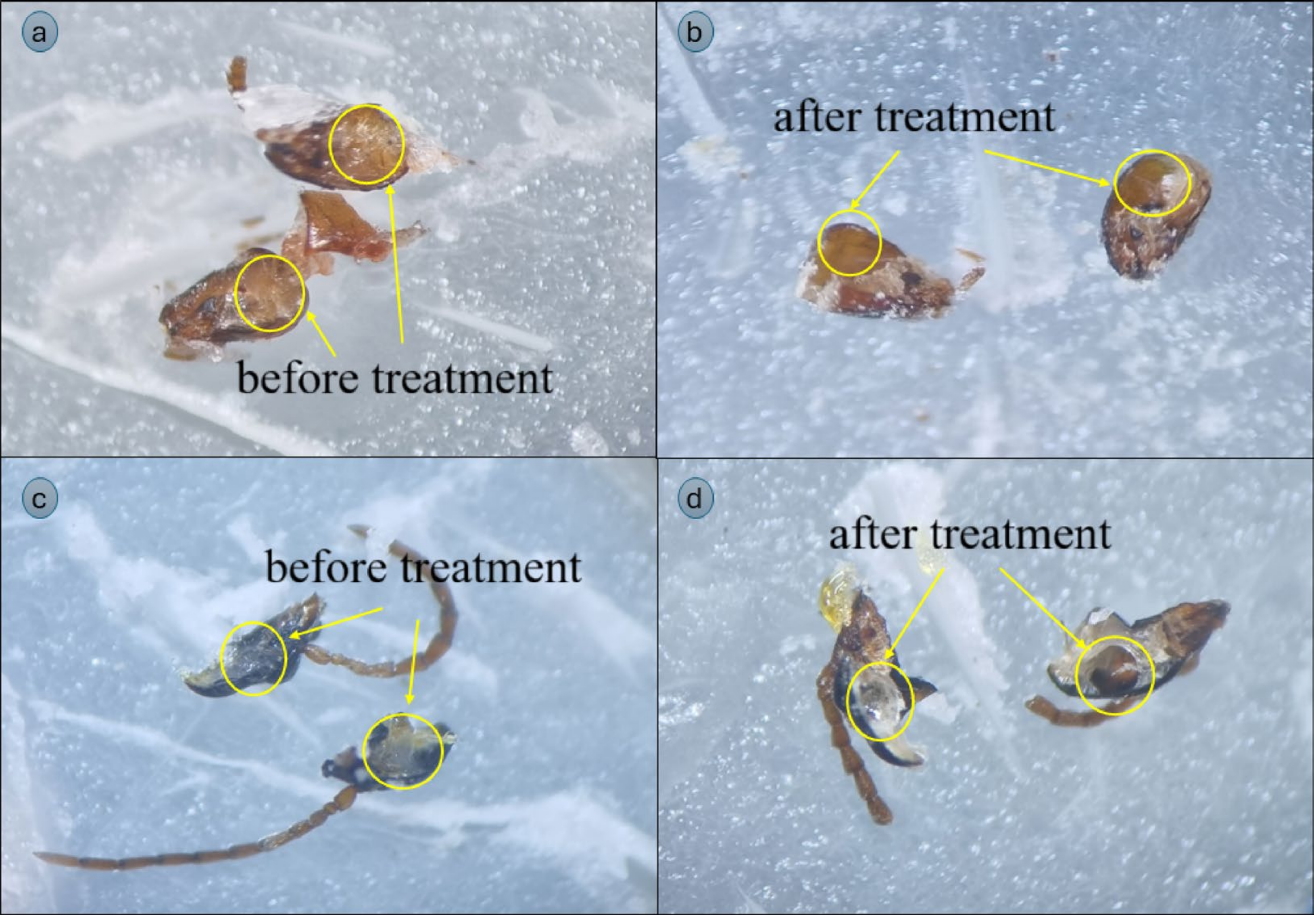
Head cross-sections of insects before and after cold plasma treatment. (**a**) *Tribolium castaneum* control: complete head structure; (**b**) *T. castaneum* treated: visibly empty internal cavity; (**c**) *Callosobruchus maculatus* control: intact structure; (**d**) *C. maculatus* treated: head shows localized rupture or hole. These anatomical damages indicate plasma-induced internal tissue breakdown.

The anatomical effects of cold plasma on insect morphology were examined through longitudinal head sections of *Tribolium castaneum* and *Callosobruchus maculatus*. In the control specimens, both species exhibited intact, structurally dense head cavities (Fig. 4a and c), with clearly defined internal tissues and no visible degradation. In contrast, the plasma-treated insects showed internal damage. The head cavity in T. castaneum (Fig. 4b) appeared partially empty, suggesting extensive tissue degradation or collapse. At the same time, in *C. maculatus* (Fig. 4d), a distinct hole was observed within the cranial structure, indicating localized destruction or membrane rupture.

### The effect of plasma on seeds

#### Germination percentage

In the evaluation of germination percentage in chickpea varieties (Saeed and Mansour), the maximum observed difference between plasma-treated samples and the control were minimal, with a maximum deviation of approximately 2%, which was not statistically significant according to one-way ANOVA (*p* > 0.05; Tables 1 and 2). These results demonstrate that plasma treatment did not significantly affect germination percentage in either chickpea variety.

For lentil seeds, most plasma treatments did not result in statistically significant changes in germination percentage compared to the control (*p* > 0.05). However, the treatment at 22 kV for 5 min showed a significant reduction in germination percentage (12% decrease, *p* < 0.05) (Table 3). This suggests that the effect of plasma on lentil germination was highly specific to certain treatment parameters rather than systematic, with significant changes were observed only under specific exposure conditions.

#### Germination rate

Cold plasma treatment significantly altered the germination rate of all tested seed types (*p* < 0.05). The effect, however, was highly dependent on the cultivar and treatment parameters. The Saeed chickpea variety and lentil seeds showed significant positive responses under optimal conditions. For Saeed, the germination rate increased by up to 21% compared to the control, with the maximum enhancement observed at 24 kV for 9 min (Table 2). Lentil seeds exhibited an even greater maximum increase of 26% under the same treatment parameters (Table 4). In contrast, the Mansur chickpea variety showed a more limited and variable response. The most notable improvement was a 7% increase at 20 kV for 3 min (*p* < 0.05; Table 3).

Significant reductions in germination rate occurred under non-optimal plasma conditions. The most pronounced decrease was observed in Saeed seeds, which showed a 23% reduction at 20 kV for 7 min (*p* < 0.05; Table 2). The Mansur variety and lentil seeds also experienced significant reductions of 16% (20 kV, 5 min) and 17% (24 kV, 3 min), respectively (Tables 3 and 4).

Overall, the germination rate followed a non-linear, dose-dependent pattern. The results indicate that while optimized plasma exposure can stimulate germination, both insufficient and excessive treatment can significantly impair seed viability.

#### Shoot length and root length of the seedlings

Cold plasma treatment significantly altered seedling growth parameters, with effects varying non-linearly with voltage and exposure time (*p* < 0.05). Shoot length showed an overall increasing trend at 24 kV, but followed a biphasic (increase-then-decrease) pattern at lower voltages (20 and 22 kV). Root length exhibited a pronounced biphasic and dose-dependent response across all voltage levels, indicating a high sensitivity to treatment intensity.

Under optimized conditions, plasma exposure significantly enhanced growth in both chickpea varieties (*p* < 0.05). For the Saeed variety, the maximum increases were 20% for root length (22 kV, 1 min) and 26% for shoot length (22 kV, 9 min) (Table 2). The Mansur variety showed even greater enhancements, with 22% and 25% increases in root and shoot length, respectively, at 24 kV for 9 min (Table 3). Lentil seedlings exhibited more modest but significant improvements, with root and shoot lengths increasing by 12% and 11% (Table 4).

However, non-optimal treatments caused significant growth inhibition. In the Saeed variety, root length declined by 47% after prolonged exposure at 22 kV for 7 min, while shoot length decreased by 40% at 20 kV for 3 min (Table 2). The Mansur variety was most affected at 20 kV, with root and shoot lengths reduced by 44% (7 min) and 49% (9 min), respectively (Table 3). For lentil, the maximum reductions were 51% for shoot length (20 kV, 5 min) and 28% for root length (24 kV, 7 min) (Table 4).

These results demonstrate that cold plasma has a biphasic, dose-dependent effect on early seedling growth. Precise optimization of treatment parameters is crucial, as it can significantly enhance elongation, whereas overexposure leads to substantial stress-related suppression.

#### Wet and dry weight of the seedlings

Cold plasma treatment significantly altered seedling biomass in a voltage- and exposure-time–dependent manner (*p* < 0.05). Wet weight showed a general increasing trend at 24 kV but followed a biphasic response at 20 and 22 kV, where stimulation and inhibition depended on exposure duration.

In the Saeed variety, wet weight showed a maximum increase of 12% at 22 kV for 9 min, while the most significant reduction (33%) occurred at 20 kV for 5 min (*p* < 0.05; Table 2). The Mansur variety exhibited a stronger response, with wet weight increasing by ~ 25% at 24 kV for 9 min but decreasing by 26% at 20 kV for 9 min (Table 3). For lentil, the response was more limited, with only a 6% increase at 24 kV for 9 min and a 31% reduction at 20 kV for 5 min (Table 4).

Dry weight also exhibited a pronounced biphasic, dose-dependent response. In the Saeed variety, dry weight increased by a maximum of 7% (22 kV, 9 min) but was reduced by up to 25% (20 kV, 1 min) (Table 2). The Mansur variety showed a 19% increase at 24 kV for 9 min and a 44% decrease at 20 kV for 3 min (Table 3). Lentil seedlings mirrored their wet weight response, with a maximum 6% increase and a 31% decrease under the same conditions (20 kV, 5 min) (Table 4).

These findings confirm that cold plasma has a dual, dose-dependent effect on seedling biomass. While optimized treatment significantly enhances biomass, non-optimal parameters induce substantial reduction, underscoring the critical need for precise optimization of voltage and exposure time.

**Table 2:** Effect of cold plasma on germination of chickpea (saeed variety).

Saeed verity	Control	Lowest increase compared to the control	Highest increase compared to control
Germination percentage (%)	98.33^a^±1.67	96.67^a^±1.67 20 kV,3 min	100 20,22 kV;1,5,7,9 min 24 kV;5,7,9 min
Germination rate	24.77^c^±1.06	19.25^b^±1.3 20 kV,7 min	31.03^a^±1.01 24 kV,9 min
Shoot lengths of seedlings (cm)	2.34^ab^±0.26	1.46^b^±0.7 20 kV,3 min	3.13^a^±0.7 22 kV,9 min
Root length of seedlings (cm)	5.12^ab^±0.35	2.73^c^±0.14 22kV7min	6.37^a^±0.48 22 kV,1 min
Wet weight of seedlings (g)	0.2083^b^±0.01	0.1407^c^±0.02 20 kV,5 min	0.2345^a^±0.03 22 kV,9 min
Dry weight of seedlings (g)	0.0339^a^±0.0029	0.0257^b^±0.001 20 kV,1 min	0.0364^a^±0.0006 22 kV,9 min

*Data are presented as a mean value ± standard error. Different letters above bars indicate statistically significant differences among treatments according to one−way ANOVA (*p* < 0.05).

**Table 3:** Effect of cold plasma on germination of chickpea (mansur variety).

Mansur verity	Control	Lowest increase compared to the control	Highest increase compared to control
Germination percentage (%)	98.33^a^±1.67	96.67^a^±1.67 20 kV;1 min	100 20 kV;5,7 min, 22 kV;1,7 min 24 kV;9 min
Germination rate	32.36^b^±0.5	27.335^c^±0.29 20 kV,5 min	34.65 ± 0.75 ^a^±0.17 20 kV,3 min
Shoot lengths of seedlings (cm)	1.45^b^±0.11	0.75^c^±0.28 20 kV,9 min	2.92^a^±0.21 24 kV,9 min
Root length of seedlings (cm)	7.3^a^ ± 0.1	4.06^c^±0.49 20 kV,7 min	9.28^a^±0.35 24 kV,9 min
Wet weight of seedlings (g)	0.1943^b^±0.007	0.1445^b^±0.033 20 kV,9 min	0.2585^a^±0.027 24 kV,9 min
Dry weight of seedlings (g)	0.0365^b^±0.006	0.0205^b^±0.005 20 kV,3 min	0.0448^a^±0.003 24 kV,9 min

*Data are presented as a mean value ± standard error. Different letters above bars indicate statistically significant differences among treatments according to one-way ANOVA (*p* < 0.05).

**Table 4:** Effect of cold plasma on germination of lentil (Bileh Sawar).

Lentil	Control	Lowest increase compared to the control	Highest increase compared to control
Germination percentage (%)	100	88.33^b^±1.67 22 kV,5 min	100 20 kV,1,3,5,7,9 min 22 kV,1,9 min, 24 kV,1 min
Germination rate	21.52^b^±0.69	17.94^c^±0.21 24 kV,3 min	29.05^a^±0.18 24 kV,9 min
Shoot lengths of seedlings (cm)	6.51^ab^±0.14	3.23^b^±0.32 20 kV,5 min	7.26^a^±0.12 24 kV,9 min
Root length of seedlings (cm)	7.05^b^±0.24	5.13^c^±0.11 24 kV,7 min	7.94^a^±0.17 22 kV,9 min
Wet weight of seedlings (g)	0.0677^a^±0.0035	0.0468^b^±0.0032 20 kV,5 min	0.072^a^±0.0012 24 kV,9 min
Dry weight of seedlings (g)	0.0082 ^a^±0.0001	0.0064^a^±0.0001 20 kV,5 min	0.0089^a^±0.0004 24 kV,9 min

*Data are presented as a mean value ± standard error. Different letters above bars indicate statistically significant differences among treatments according to one−way ANOVA (*p* < 0.05).

### FTIR analysis of plasma-treated seeds and substrates

**Fig. 5:**
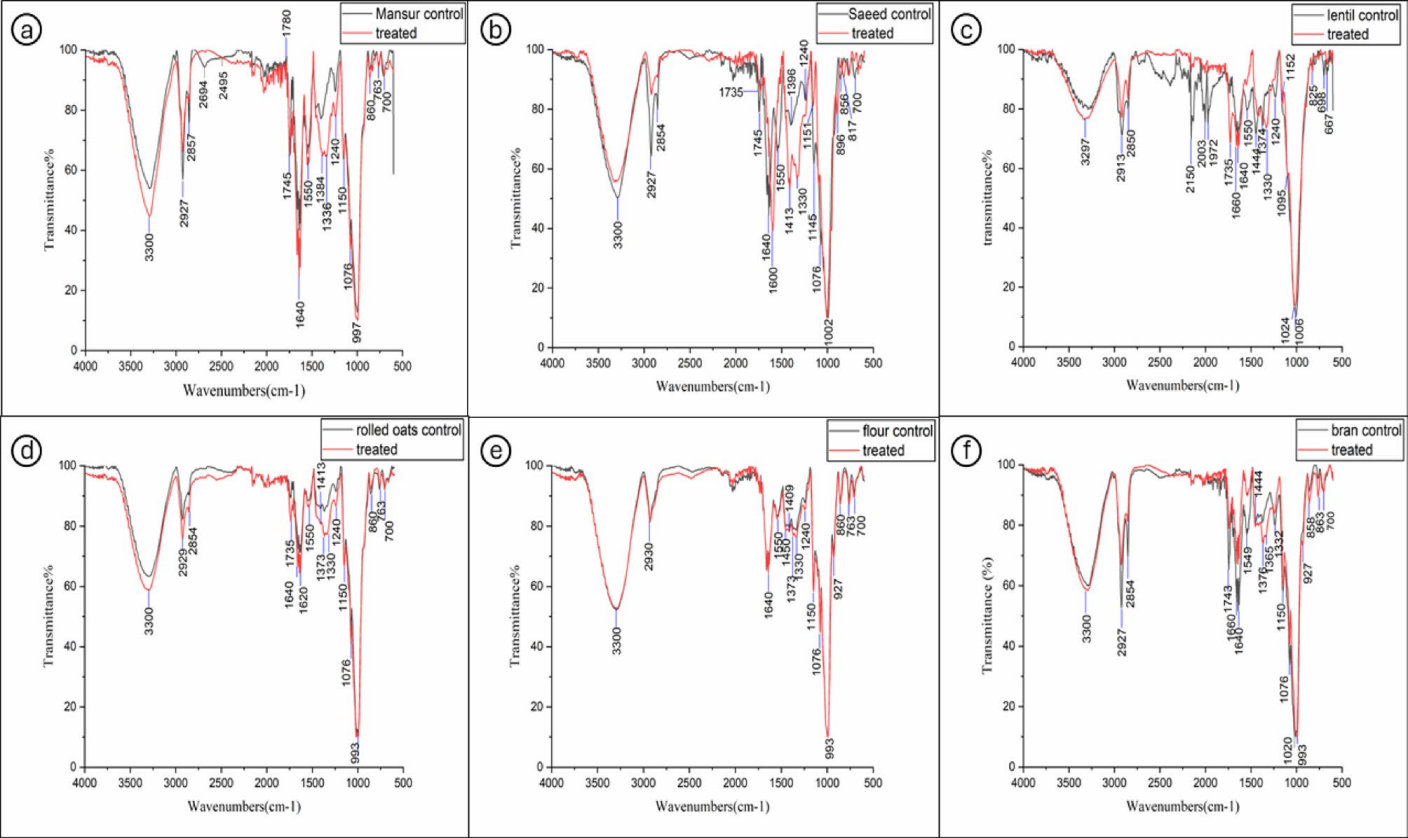
FTIR spectra of control (black) and plasma-treated (red) samples of chickpea (Mansur (**a**) and Saeed (**b**)), lentil (**c**), rolled oats (**d**), flour (**e**), and bran (**f**). The O–H/N–H stretching region (~ 3200–3500 cm⁻¹) and fingerprint region (~ 1100–1450 cm⁻¹) show significant intensity and peak broadening increases, indicating enhanced hydrophilicity due to surface functionalization.

FTIR spectroscopy was used to investigate chemical surface modifications in seeds and substrates following cold plasma treatment, focusing on water absorption–related functional groups (Fig. 5a-f). Particular attention was given to the O–H and N–H stretching region (~ 3200–3500 cm⁻¹) and the fingerprint region (~ 1100–1450 cm⁻¹), which revealed significant structural changes after treatment. Except for flour, in all samples—lentil, chickpea (Saeed and Mansur), bran, and rolled oats—the spectra of plasma-treated materials (red lines) showed clear increases in intensity and broadening of bands in both targeted regions compared to untreated controls (black lines). In the 3200–3500 cm⁻¹ region, treated samples showed intensified and broadened O–H and N–H stretching bands, indicating enhanced surface hydrophilicity due to the introduction of polar groups. This effect was most potent in the rolled oats and chickpea variety Mansur, reflecting greater chemical surface activation.

In addition, consistent and significant changes were observed in the 1100–1450 cm⁻¹ region:

- Around 1450–1430 cm⁻¹, increased C–H bending vibrations (CH₂/CH₃) were noted, suggesting lipid breakdown or rearrangement on the seed surfaces.
- Enhanced signals at 1400–1370 cm⁻¹ indicate carboxylate symmetric stretching (–COO⁻), likely resulting from oxidation of amino acid side chains or surface proteins.
- Bands near 1330–1250 cm⁻¹ reflect C–N stretching and N–H bending (amide III region), suggesting modification of seed proteins through plasma-induced denaturation or hydrolysis.
- Finally, the substantial enhancement of bands between 1150 and 1000 cm⁻¹ corresponds to C–O and C–O–C stretching, which are typical for polysaccharides like cellulose, hemicellulose, and starch.

These shifts indicate that plasma disrupted and oxidized carbohydrate structures, making their functional groups more IR-active and accessible^32–34^.

### SEM analysis of plasma-treated seeds

**Fig. 6:**
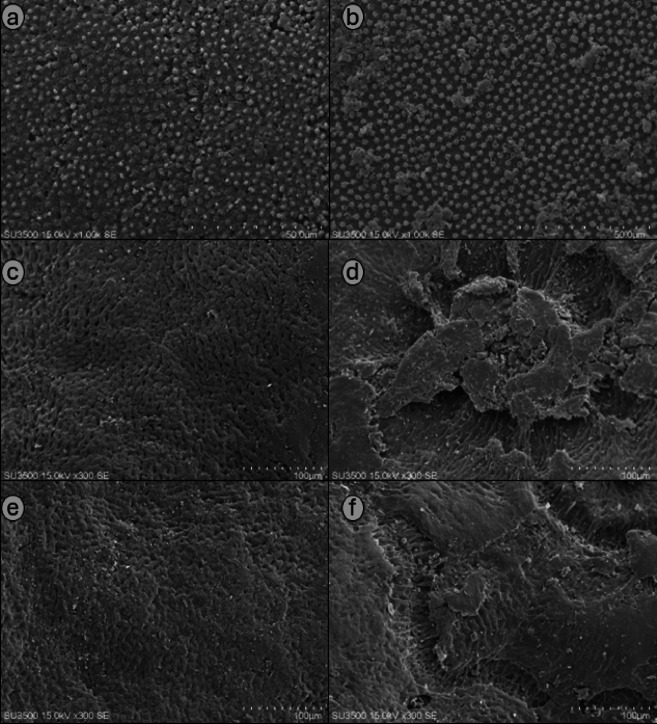
Scanning electron microscopy (SEM) images of lentil (Bileh Sawar) (**a**,**b**) and chickpea (Mansur (**c**, **d**) and Saeed (**e**, **f**)) seed surfaces before (control) and after cold plasma treatment. Control seeds displayed smooth, intact surfaces, whereas treated seeds exhibited visible shrinkage and surface etching, indicating plasma-induced desiccation and oxidative erosion—scale bars: 100 μm for chickpeas; 50 μm for lentil.

SEM imaging revealed consistent morphological alterations across all three tested seed types—lentil, and the Saeed and Mansur chickpea varieties—following cold plasma exposure. In the untreated control samples, the seed coats exhibited smooth, dense, and compact surface structures, characteristic of intact protective outer layers that minimize water permeability and mechanical stress (Fig. 6a, c,e). After treatment, however, the SEM micrographs showed marked shrinkage, wrinkling, and surface collapse in all three cases (Fig. 6b, d,f). In lentil seeds, the surface appeared severely deformed, with visible compression and loss of epidermal tension. Similar patterns were observed in Saeed and Mansur chickpeas, with the outer layers showing clear contraction and disruption, indicating breakdown of surface integrity.

### Contact angle measurements and surface wettability

**Fig. 7:**
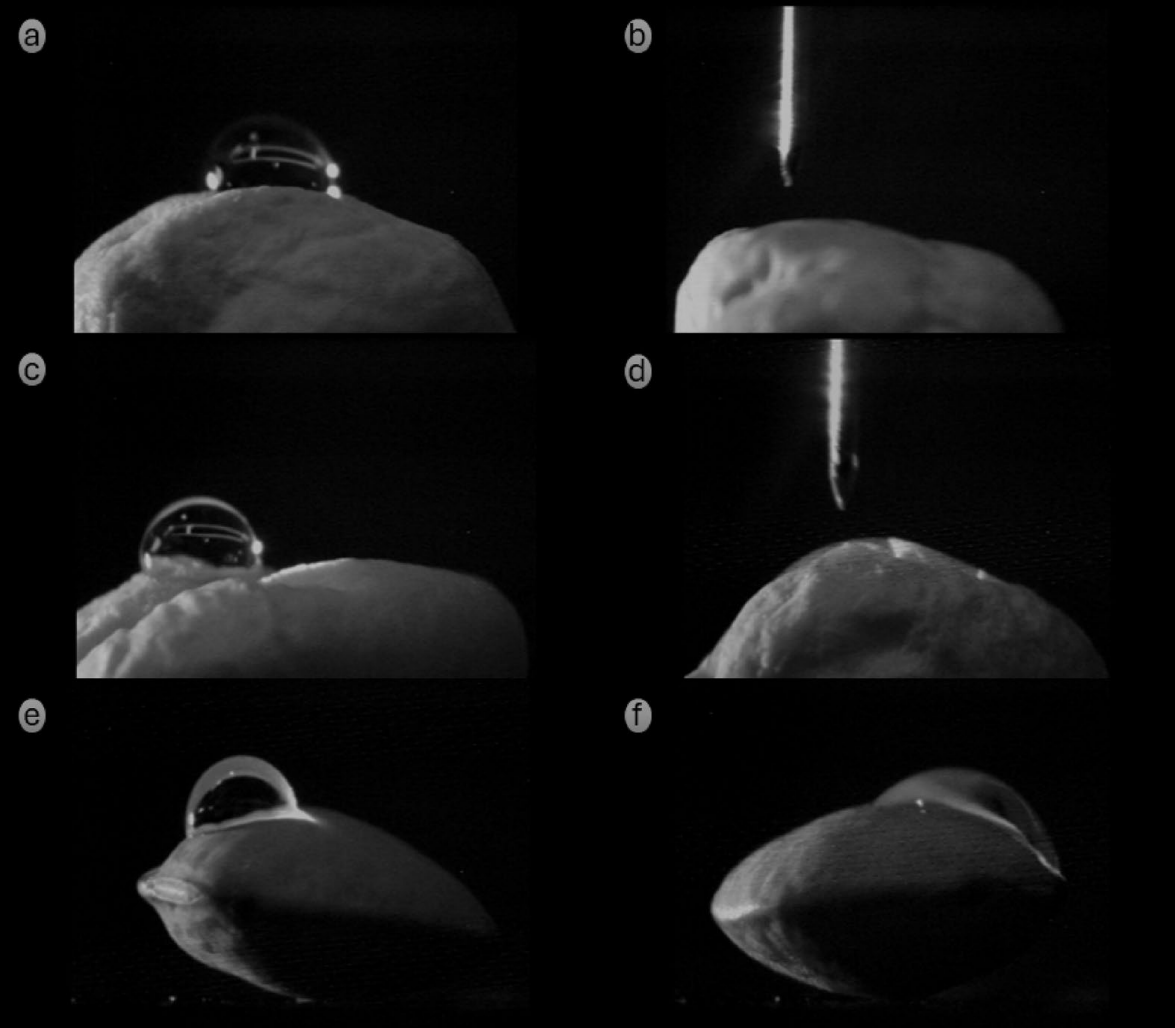
Contact angle images of seeds before and after cold plasma treatment. (**a**) Saeed variety control (75.57º); (**b**) Saeed variety treated (0º); (**c**) Mansur variety control (80.02º); (**d**) Mansur variety treated (11.92º); (**e**) Lentil control (86.35º); (**f**) Lentil treated (25.73º). Treated samples exhibit visibly reduced contact angles, indicating enhanced hydrophilicity and improved water absorption.

Contact angle analysis provided visual and quantitative confirmation of the enhanced hydrophilicity of seed surfaces following cold plasma treatment. In the untreated control samples—Saeed, Mansur, and lentil—the water droplets remained nearly spherical, maintaining high contact angles that reflect low surface wettability and resistance to water absorption (Fig. 7a, c, and e). This hydrophobic behavior is typical of seed coats containing intact lipids, wax, and cutin layers that function as water barriers. In contrast, the plasma-treated seeds exhibited significantly reduced contact angles (Fig. 7b, d, and f), with droplets visibly spreading over the surface. This transformation indicates a clear shift toward hydrophilic surface properties, consistent with increased water-absorption capacity. The improvement in wettability can be attributed to chemical and physical modifications induced by plasma, including the oxidation of surface groups, the formation of polar functional moieties (e.g., hydroxyl and carboxyl groups), and increased surface roughness^35^. The consistency of this effect across all three seed types confirms that cold plasma treatment effectively increases surface energy and water affinity, supporting the FTIR and SEM findings. This property is particularly advantageous for promoting faster water uptake during germination, which is often a limiting factor for seed vigor and early growth performance^36^.

### Plasma species characterization and their functional roles

**Fig. 8:**
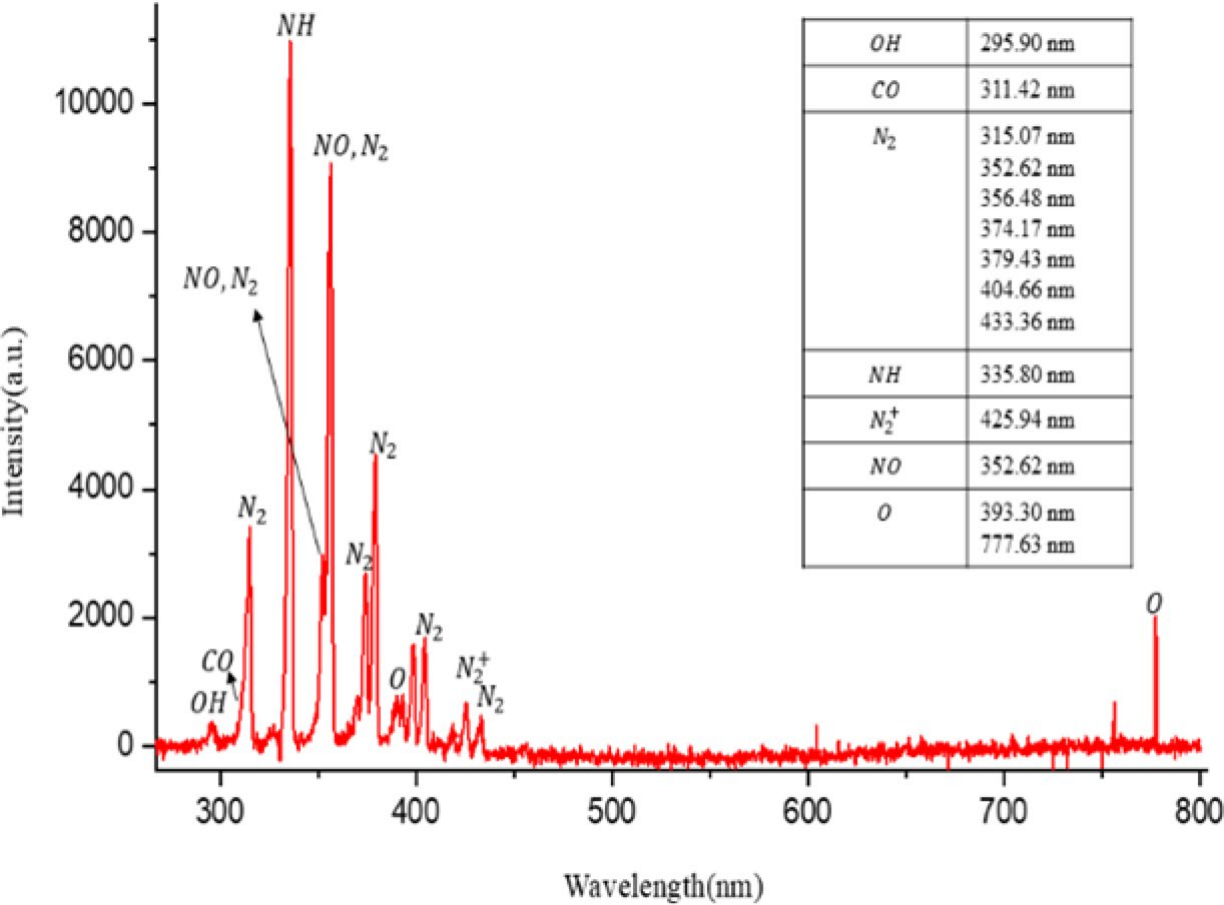
Optical emission spectroscopy (OES) of the dielectric barrier discharge (DBD) plasma used in this study. Prominent peaks were identified using the NIST database and correspond mainly to reactive nitrogen species (N₂, N₂⁺), as well as hydroxyl radicals (OH) and nitric oxide (NO). The abundance of reactive oxygen and nitrogen species (ROS/RNS) confirms their central role in surface modification, oxidative stress induction, and the biological effects observed on insects and seed germination.

Optical emission spectroscopy (OES) was employed to characterize the chemical species in the plasma used in this experiment. Spectral measurements were carried out using an Avantes spectrometer. The plasma generated was accessed through a viewing window on the plasma chamber for data acquisition. The spectrometer’s optical fiber was positioned directly in front of this window to capture the emitted light, and the resulting plasma spectrum was recorded (Fig. 8). Peak identification was performed by referencing the National Institute of Standards and Technology (NIST) atomic spectra database and by comparison with previous plasma studies. The analysis confirmed that the discharge contained a variety of reactive oxygen species (ROS) and reactive nitrogen species (RNS). Notably, the majority of the detected emission peaks corresponded to excited nitrogen species, which are known to contribute significantly to both surface modification and biological effects during plasma treatment^37^.

## Discussion

The present study demonstrates that cold plasma treatment exerts significant, life stage–dependent insecticidal effects, underscoring its potential as a physical pest management tool for stored products. Treatment induced rapid and substantial mortality in both Callosobruchus maculatus and Tribolium castaneum, though the specific response varied between species and developmental stages. In C. maculatus, adult mortality exceeded 40% immediately after treatment in all plasma-exposed groups and reached 100% under several voltage–time combinations (e.g., 24 kV for ≥ 5 min), demonstrating a potent and immediate lethal effect. Exposure duration emerged as a more critical factor than applied voltage for achieving lethality, a trend consistent with previous reports on plasma-based insect control^25,38,39^. Beyond direct mortality, plasma treatment profoundly suppressed adult emergence from eggs. While control samples reached 100% emergence within six days, emergence in treated groups was significantly lower, with complete inhibition (0%) at 24 kV for 9 min. This suppression was strongly dependent on both voltage and exposure time, showing a clear dose-response relationship. These results align with earlier findings for C. chinensis eggs, where plasma was proposed to disrupt the chorion, impairing gas exchange and preventing development^5^. For T. castaneum, plasma susceptibility varied markedly among life stages, defining a clear hierarchy of sensitivity. Larvae were the most vulnerable, with mortality exceeding 90% after only 5 s at 23 kV. Adults were less sensitive, requiring 30 s at 23 kV to achieve 100% mortality. Pupae exhibited the greatest resistance, needing 90 s at 23 kV for complete mortality. This stage-dependent resistance profile is consistent with entomological principles; the thicker, more sclerotized pupal cuticle^40^ and reduced metabolic activity during this developmental transition^41^ likely confer higher tolerance to plasma-induced oxidative stress.

The insecticidal efficacy observed here aligns closely with the body of research supporting cold plasma as a viable alternative to chemical pesticides. Our findings on C. maculatus corroborate the work of Pathan et al., who reported effective control of C. chinensis in stored chickpea using plasma^5^, Similarly, the positive correlation between mortality and treatment intensity (voltage/time) for T. castaneum adults matches the results of Radhakrishnan et al.^25^. The distinct sensitivity of larvae compared to adults and pupae further validates the stage-specific effects reported by Ziuzina et al.^8^. Notably, while Hassan et al. achieved similar efficacy using radio-frequency plasma with inert carrier gases^39^. our study attained comparable results with an air-based DBD system, highlighting a practical and cost-effective advantage. Furthermore, the absence of insect recovery 24 h post-treatment in our study suggests the inflicted damage was irreversible, a crucial factor for reliable pest control that contrasts with some reports of sublethal effects and recovery^42^.

Previous studies have reported various effects of plasma on insect bodies. Donohue et al. reported that plasma exposure causes damage to the nervous and/or neuromuscular systems in *cockroaches*. In a way, they lost their photo-, vibro-, and thigmo-tropic responses and were unable to right themselves^38^. Abd El-Aziz et al. also found that plasma treatment can cause malformations in treated larvae, pupae, or emerging adults of *Plodia Interpunctella*^1^. Our study’s microscopic examination of head sections revealed apparent structural damage in both species, including tissue collapse and localized ruptures. Such degradation likely results from oxidative and structural disruption of the cetin and protein matrices by ROS/RNS, along with localized heating and dehydration^28^. Further studies are needed to fully elucidate the mechanisms underlying plasma-induced developmental arrest.

Cold plasma treatment elicited a biphasic, dose-dependent influence on seed germination and early seedling development, with outcomes varying significantly by species and cultivar. The final germination percentage remained high and statistically unchanged for both chickpea varieties (Saeed and Mansur), indicating robust tolerance. In contrast, lentil seeds exhibited a significant reduction (12%) under a specific, non-optimal condition (22 kV, 5 min) (*p* < 0.05). This differential sensitivity likely stems from variations in seed coat composition and permeability, which modulate the penetration of reactive plasma species and the subsequent oxidative stress. This aligns with the findings of Dhayal et al., who correlated greater plasma-induced oxidative damage with thinner seed coats^42^. However, the predominant maintenance of high viability in our study suggests a more favorable therapeutic window for the applied plasma parameters compared to earlier reports. Germination rate and seedling vigor displayed clear optimization curves. Significant enhancements were achieved under specific conditions: the Saeed chickpea and lentil showed maximum germination rate increases of 21% and 26%, respectively, at 24 kV for 9 min. The Mansur variety responded more modestly, with a peak increase of 7% (20 kV, 3 min). These cultivar-specific optima underscore the importance of tailored treatment protocols and resonate with the work of Sayahi et al., who documented a wide range of plasma responsiveness among soybean cultivars^43^.

This trend continued in seedling growth metrics. Optimal plasma exposure significantly promoted elongation, with chickpea varieties achieving > 20% increases in shoot and root length. Lentil seedlings showed smaller but significant improvements (11–12%). These results corroborate numerous studies reporting plasma-mediated enhancement of early plant growth^44–46^, such as the 37% increase in black gram seedling growth observed by Billah et al. Critically, however, the beneficial effects were tightly constrained by dosage. Suboptimal or excessive treatment led to severe growth inhibition, with root and shoot length reductions exceeding 40% in some cases. This sharp decline is consistent with plasma-induced oxidative damage surpassing cellular repair capacities, disrupting vital processes like cell division and elongation^47,48^. Biomass data reinforced this dual, dose-dependent paradigm. Maximum wet weight gains of 25% (Mansur chickpea) were recorded under optimal settings (24 kV, 9 min), while the most detrimental treatments caused losses exceeding 30% (e.g., lentil at 20 kV, 5 min). This pattern mirrors findings in cereals like wheat and oats^49^, and conclusively demonstrates that cold plasma acts as a potent biostimulant or a stressor, with the outcome dictated by a precise interplay between treatment parameters and inherent seed physiology.

There appears to be an indirect relationship between seed germination and water uptake. Adequate hydration is a critical factor in initiating the metabolic processes required for germination, and any modification to the seed surface that enhances water permeability can accelerate this process. Several studies have reported that plasma treatment can improve seed germination and seedling vigor by increasing surface wettability and altering the physicochemical properties of the seed coat, thereby facilitating greater water absorption^50,51^. Yamauchi et al. reported that plasma treatment of Sophora flavescens seeds accelerated swelling due to enhanced water absorption. They further noted that swelling and subsequent seed coat rupture represent a critical stage in germination, as coat rupture facilitates root emergence and thereby accelerates germination and seedling growth^52^.

To explain physiological trends, we examined chemical and structural modifications at the seed surface using FTIR, SEM, and contact angle analysis. FTIR spectra revealed significant alterations in functional groups, particularly in the O–H and N–H stretching regions (3200–3500 cm⁻¹) and in the C–O and C–N stretching regions (1100–1450 cm⁻¹). These changes suggest the introduction of polar oxygen- and nitrogen-containing groups, which increase seed coat hydrophilicity^24^. In lentil, marked differences were also seen in the 1700–2800 cm⁻¹ region, corresponding to C–H stretching vibrations, where treated seeds exhibited altered peak intensities, indicating modifications to lipid and protein structures. Such chemical changes facilitate faster water absorption by disrupting hydrophobic barriers and loosening cross-linked structures, including carboxyl groups whose partial breakdown enhances permeability and uptake^33^. SEM imaging provided direct evidence of these changes. While untreated seeds displayed smooth, intact surfaces, plasma-treated seeds showed visible shrinkage and etching of the outer layer. This shrinkage likely results from localized desiccation, ablation, and oxidative breakdown of the cuticle caused by energetic ion bombardment and reactive species^53,54^ such as oxygen and nitrogen species (ROS/RNS), including OH, NO, and excited N₂ species, as indicated by optical emission spectroscopy (OES). The altered surface microstructure increased porosity and, combined with the chemical modifications revealed by FTIR, explains the enhanced water uptake observed in contact angle experiments. Hence, Wang et al. reported that the partial degradation of surface compounds and formation of cracks in the cell outer layer of cotton seeds significantly enhanced their water absorption capacity, as evidenced by the rapid sinking of treated seeds in water, whereas untreated control seeds remained afloat^34^.

Taken together, our results demonstrate that the positive effects of cold plasma on germination and early growth arise from a combination of physical surface etching, chemical oxidation of the seed coat, and the biological action of reactive plasma species. By integrating morphological (SEM), chemical (FTIR), and wettability (contact angle) analyses, we provide direct evidence that plasma modifies the structure and chemistry of seed surfaces, thereby regulating water uptake and seedling vigor. These mechanistic insights help explain the species- and variety-specific responses observed in our germination experiments and underscore the importance of carefully balancing plasma parameters to maximize benefits while minimizing stress.

Despite the promising outcomes observed in this study, several limitations should be acknowledged. The biological effects of cold plasma were highly dependent on treatment parameters, species, and developmental stage, indicating that a single plasma condition cannot be universally applied for both insect control and seed enhancement. Moreover, excessive exposure durations or non-optimal voltage levels resulted in inhibitory effects on seed germination and seedling growth, emphasizing the importance of precise optimization. In addition, the experiments were conducted under controlled laboratory conditions, and further studies are required to evaluate the long-term effects of plasma treatment during storage and under field-relevant conditions. Future work should also focus on scaling up plasma systems and elucidating the molecular mechanisms underlying plasma–biological interactions.

## Conclusion

This study demonstrated that cold plasma treatment exhibits dual-functionality, serving as a promising, eco‑friendly approach that can enhance seed performance and provide effective pest control under optimized laboratory conditions. Our results show that optimal plasma parameters significantly improved germination rate, seedling growth, and biomass in chickpea (Saeed and Mansur) and lentil, with enhanced surface hydrophilicity and structural modifications—confirmed by FTIR, SEM, and contact angle analyses—helping to explain these improvements. Concurrently, plasma exposure caused substantial, stage‑specific mortality in Callosobruchus maculatus and Tribolium castaneum, accompanied by observable morphological damage to insect tissues. Optical emission spectroscopy confirmed the generation of reactive oxygen and nitrogen species, which are likely contributors to the observed biological effects. Together, these findings underscore the potential of cold plasma as a sustainable pre‑sowing and pest‑management tool. However, the strong dependence of outcomes on treatment parameters highlights that precise voltage and exposure‑time optimization is critical to maximize benefits and avoid detrimental effects. Further research is needed to scale the technology for commercial storage and field applications.

## Data Availability

The data sets used and analyzed during the current study are available from the corresponding author upon reasonable request.
